# Oral Rhabdomyomatous Hamartoma in a Paediatric Patient: Case Report a Rare Lesion

**DOI:** 10.1111/ipd.70138

**Published:** 2026-08-09

**Authors:** Érika Machado do Carmo Albuquerque, Letícia Albuquerque Rodrigues, Gislayne Nunes de Siqueira, Marcelo Bonifácio da Silva Sampieri, Filipe Nobre Chaves, Denise Hélen Imaculada Pereira de Oliveira

**Affiliations:** ^1^ Postgraduate Program in Health Sciences Federal University of Ceará – Sobral Campus Sobral Brazil; ^2^ Postgraduate Program in Pathology Federal University of Ceará Fortaleza Brazil

**Keywords:** case report, differential diagnosis, hamartoma, oral cavity, paediatric dentistry

## Abstract

**Background:**

Rhabdomyomatous hamartoma is a rare congenital lesion, usually described on the face and neck and infrequently found in the oral cavity. Because its clinical presentation is non‐specific, histopathological examination plays a fundamental role in establishing the final diagnosis and determining appropriate therapeutic management.

**Case Report:**

An 8‐year‐old male patient presented with a submucosal nodule on the left lateral border of the tongue. The initial diagnostic hypothesis was dermoid cyst; however, histopathological examination revealed thick, interlacing bundles of skeletal striated muscle, compatible with rhabdomyomatous hamartoma.

**Conclusion:**

Although rare, rhabdomyomatous hamartoma should be considered in the differential diagnosis of oral nodules in children. Histopathological confirmation is essential for accurate diagnosis and as a guide to treatment, helping to avoid unnecessary interventions and ensuring proper management of the lesion.

## Why This Paper Is Important to Pediatric Dentists

- This article reinforces the importance of including rhabdomyomatous hamartoma in differential diagnoses of oral nodules in children.
- The article emphasizes that histopathological examination is essential for accurate diagnosis thereby avoiding unnecessary interventions.
- The article demonstrates that early recognition of RH facilitates appropriate management and timely referral to a specialist.

## 1 Introduction

Rhabdomyomatous hamartoma (RH) is a rare congenital lesion that primarily affects the skin of the face and neck, being uncommon in the oral cavity [1, 2, 3]. Its aetiology remains unknown, but hypotheses suggest aberrant embryonic migration of mesodermal tissues or a genetic defect predisposing to the condition [2, 3]. RH is most often observed in new‐borns and children, with no gender predilection [2].

Clinically, RH may present as a solitary papule or nodule, though multiple lesions can occur and in some cases, it may be associated with congenital anomalies such as Delleman syndrome [1, 3]. Histologically, RH is characterized by proliferating bundles of striated muscle permeated by small blood vessels, collagen, and nerve fibres. These findings are critical for diagnosis [1, 2, 3].

The treatment of choice is surgical excision, and to date, recurrence has not been reported following complete excision [1, 2, 3]. Due to its rarity in the oral cavity, additional case reports are essential to expand clinical and histopathological recognition of this lesion.

## 2 Case Report

An otherwise healthy 8‐year‐old male patient presented to the stomatology clinic with a complaint of a nodule on the left lateral border of the tongue. According to the patient's caregivers, the lesion was first noticed approximately 1 month before the initial consultation and was associated with mild discomfort during feeding. Intraoral examination revealed a submucosal lesion of approximately 3 cm, with colour similar to the adjacent mucosa, firm consistency, non‐fixed to the underlying tissues, smooth surface, with a 1‐month history (Figure 1). An incisional biopsy under local anaesthesia was performed, with a primary clinical hypothesis of dermoid cyst.

**FIGURE 1:**
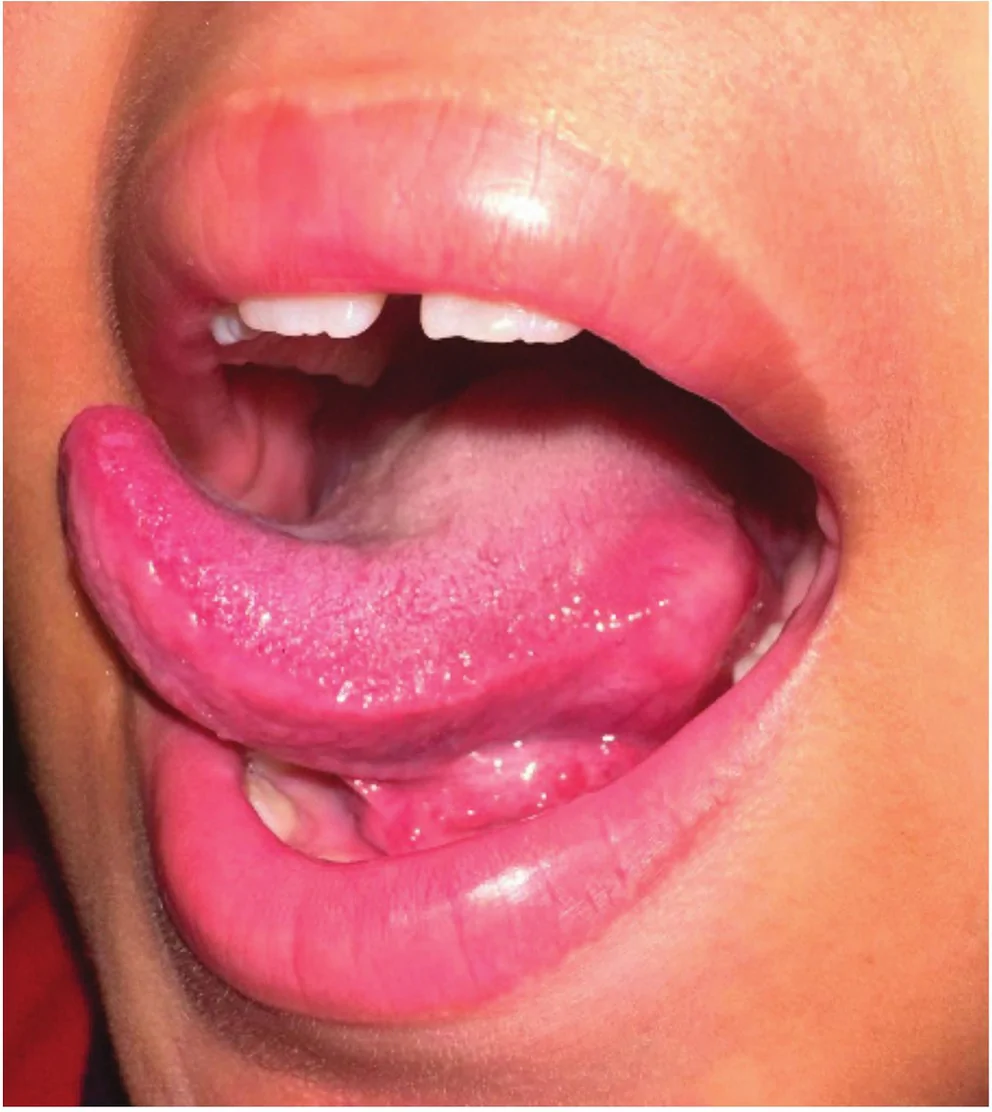
Clinical appearance of the lesion located in the posterior region of the lateral border of the tongue on the left side.

Histopathological examination revealed oral mucosa containing thick, interlacing bundles of striated skeletal muscle oriented in transverse and longitudinal sections, permeated by mature adipocytes and nerve bundles in the deeper tissue. These findings were consistent with rhabdomyomatous hamartoma (Figure 2).

**FIGURE 2:**
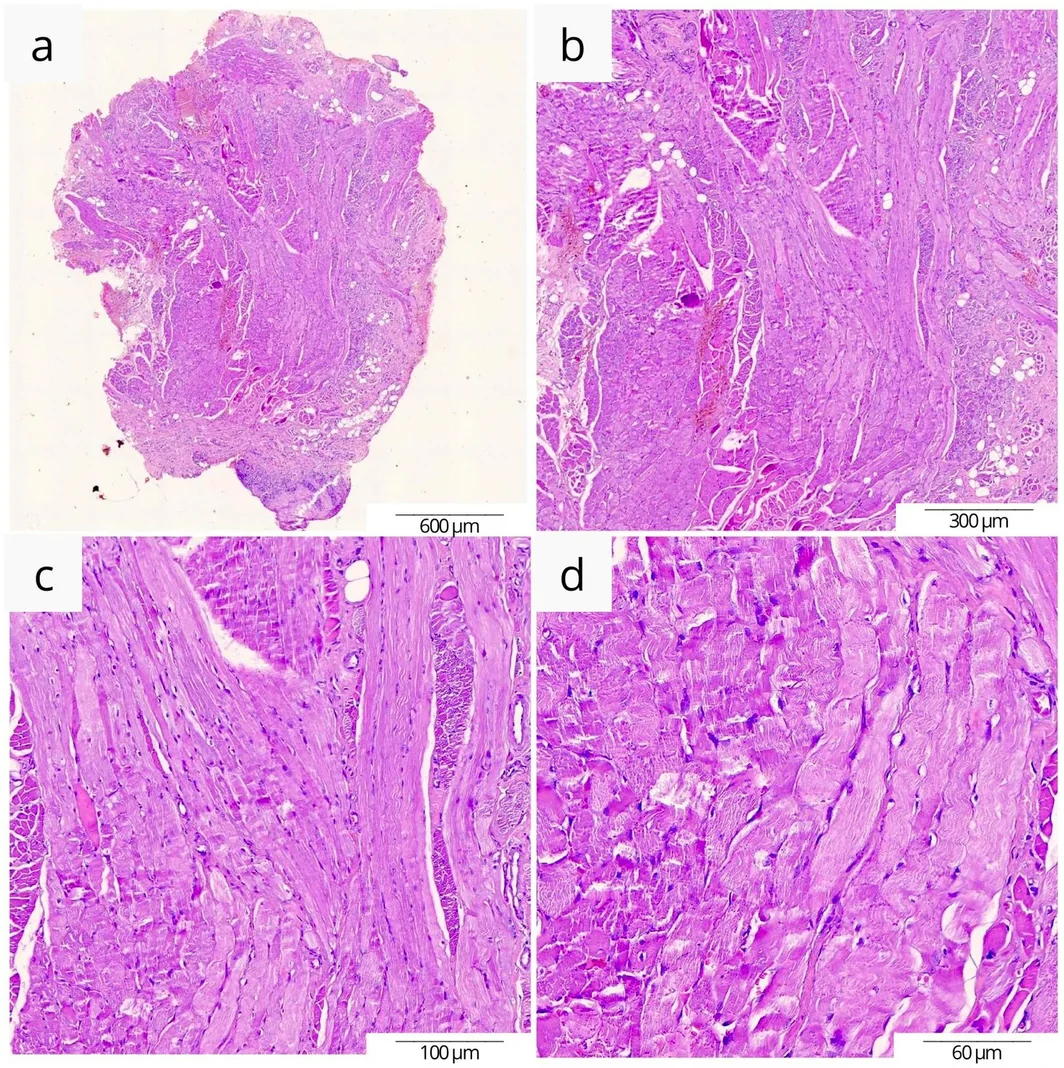
(a) At higher magnification, a fragment of oral mucosa covered by hyperplastic parakeratinized stratified squamous epithelium can be observed (scale bar = 600 μm). (b) In its depth, thick, intersecting bundles of skeletal muscle tissue are observed (scale bar = 300 μm). (c, d) Muscle bundles with longitudinal (scale bar = 100 μm) and transverse orientation (scale bar = 60 μm), respectively.

Given the lesion's location on the posterior tongue, the challenges associated with surgical management in paediatric patients, and the histopathological diagnosis of RH, the Oral and Maxillofacial Surgery team elected to manage the residual lesion conservatively with clinical follow‐up after the incisional biopsy. At follow‐up, no signs of progression of the residual lesion were observed, and periodic clinical monitoring was continued because of the potential for local trauma. Informed consent for publication was obtained from the parents.

## 3 Discussion

The term *hamartoma* is used in medical literature to describe a lesion composed of normal tissue elements that form a tumour‐like lesion consisting of a disorganized mixture of mature tissues [5]. Like other hamartomas, RH represents a disorganized proliferation of mature tissues, including mature adipose tissue, skeletal muscle, adnexal structures and occasionally blood vessels and nerves [3, 4] which form a benign lesion that can mimic congenital tumours or cysts.

RH aetiology remains unclear, but theories point to embryonic migration of mesodermal tissues or genetic factors [3, 5]. Although RH is mostly reported in the face and neck, intra‐oral involvement remains rare, with isolated cases reported in the literature [3, 4], and the tongue being one of the least frequent site [1].

Reports of RH involving the tongue are scarce in the literature [3, 4, 5]. Previously reported cases have occurred predominantly in neonates, infants, and children, presenting as benign‐appearing nodular or polypoid lesions [3, 4, 5]. Similar to these reports, the present case occurred in a paediatric patient and involved the tongue. However, its non‐specific clinical presentation initially led to a provisional diagnosis of a dermoid cyst, which highlights the challenges of establishing an accurate preoperative clinical diagnosis.

Because its clinical appearance is non‐specific, usually as a papule or solitary nodule, the diagnosis may be challenging, especially since developmental cysts, reactive hyperplasias and benign neoplasms are more commonly observed in children [1, 3, 5].

The differential diagnosis may also include dermoid cyst, vascular malformations, lingual thyroid, granular cell tumour and other hamartomas [5]. As there are no pathognomonic clinical features, histopathological examination remains essential for definitive diagnosis [4, 5], and RH should be considered in the differential diagnosis of lingual nodules in paediatric patients.

Histopathological confirmation is crucial because the presence of randomly distributed striated muscle bundles associated with adipose tissue, collagen fibres, blood vessels and elastic fibres will define the diagnosis and allow for differentiation [4, 5]. In the present case, the histopathological findings were sufficiently characteristic for the diagnosis of RH, making complementary tests, such as immunohistochemistry, unnecessary. However, in morphologically doubtful cases, immunohistochemical studies using muscle differentiation markers such as desmin, muscle‐specific actin, and myogenin may assist in confirming the diagnosis [1]. Treatment consists of complete surgical excision, and the literature presents an excellent prognosis with no reports of recurrence [2, 5].

In the case presented, the relatively large size and lingual location of the lesion highlight the importance of considering RH in the differential diagnosis to avoid inappropriate management and ensure correct referral to a specialist as needed. Additional reports contribute by broadening the clinical spectrum and help support recognition of this rare yet relevant entity in paediatric care.

Considering the rarity of rhabdomyomatous hamartoma in the oral cavity, it is unlikely to be initially considered a diagnostic hypothesis. This case adds to the existing literature and reinforces the need to include RH in the differential diagnosis of oral nodules in children while highlighting the importance of histopathological examination for appropriate therapeutic planning.

## Author Contributions

E.M.d.C.A., L.A.R. and G.N.d.S. contributed to the writing of the scientific article. M.B.d.S.S. and F.N.C. contributed guidance in conducting and documenting the clinical case. D.H.I.P.d.O. contributed guidance in conducting the clinical case, in its documentation, and also in writing and reviewing the scientific article.

## Funding

The authors have nothing to report.

## Ethics Statement

Ethical approval was not required for this case report according to institutional requirements.

## Consent

Written informed consent for publication was obtained from the patient's parents.

## Conflicts of Interest

The authors declare no conflicts of interest.

## Data Availability

Data sharing not applicable to this article as no datasets were generated or analysed during the current study.

## References

[1] A. Dal Vechio , F. R. Dal Vechio , D. A. de Oliveira , et al., “Rhabdomyo matous (Mesenchymal) Hamartoma Presenting as Haemangioma on the Upper Lip: A Case Report With Immunohistochemical Analysis and Treatment With High‐Power Lasers,” Case Reports in Dentistry 2013 (2013): 943953. 10.1155/2013/943953 PMC3679716 23781351

[2] X. B. Jiménez Polanco , S. Arias‐Santiago , J. Aneiros‐Fernández , et al., “Post‐Traumatic Lip Lesion Mimicking Rhabdomyomatous Mesenchymal Hamartoma in a Paediatric Patient,” Dermatology Online Journal 26, no. 11 (2020): 13030/qt40q9s5kd. 33342181

[3] M. Vaidyanathan , C. E. C. S. Williams , and P. R. Morgan , “Rhabdomyomatous Mesenchymal Hamartoma of the Tongue,” BMJ Case Reports 2011 (2011): bcr0820103225. 10.1136/bcr.08.2010.3225 PMC3118904 22692645

[4] G. Magro , A. Di Benedetto , G. Sanges , F. Scalisi , and R. Alaggio , “Rhabdomyomatous Mesenchymal Hamartoma of Oral Cavity: An Unusual Location for Such a Rare Lesion,” Virchows Archiv 446, no. 3 (2005): 346–347. 10.1007/s00428-004-1196-9 15726403

[5] P. D. Sotta , C. Salomão , and S. González , “Rhabdomyomatous (Mesenchymal) Hamartoma of the Tongue: Report of a Case,” Journal of Oral Pathology & Medicine 36, no. 1 (2007): 58–59. 10.1111/j.1600-0714.2006.00457.x 17181744

